# Direct interaction between RecA and a CheW-like protein is required for surface-associated motility, chemotaxis and the full virulence of *Acinetobacter baumannii* strain ATCC 17978

**DOI:** 10.1080/21505594.2020.1748923

**Published:** 2020-04-07

**Authors:** Jordi Corral, María Pérez-Varela, Jordi Barbé, Jesús Aranda

**Affiliations:** Departament de Genètica i Microbiologia, Facultat de Biociènces, Universitat Autònoma de Barcelona, Bellaterra, Spain

**Keywords:** *A. baumannii*, surface-associated motility, chemotaxis, virulence, RecA, CheW-like protein

## Abstract

*Acinetobacter baumannii* is a nosocomial pathogen that causes multi-drug resistant infections mainly in immunocompromised patients. Although this gram-negative species lacks flagella, it is able to move over wet surfaces through a not well characterized type of movement known as surface-associated motility. In this study we demonstrate through the inactivation of the *A1S_2813* gene (coding a CheW-like protein) and *recA* (coding a DNA damage repair and recombination protein) that both genes are involved in the surface-associated motility and chemotaxis of *A. baumannii* ATCC 17978 strain. In addition, we also point out that the lack of either RecA or CheW-like proteins reduces its virulence in the *Caenorhabditis elegans* and the *Galleria mellonella* animal models. Furthermore, we show through co-immunoprecipitation assays that the CheW-like protein and RecA interact and that this interaction is abolished by the introduction of the mutation S97A in one of the domains of CheW-like protein that is structurally conserved in *Salmonella enterica* and necessary for the RecA-CheW interaction in this bacterial species. Finally, we show that the replacement of the wild-type CheW-like protein by that presenting the S97A mutation impairs surface-associated motility, chemotaxis and virulence of *A. baumannii* strain ATCC 17978.

## Introduction

*Acinetobacter baumannii* is an opportunistic pathogen whose abilities to develop antimicrobial resistance and to survive in hospital environments are responsible for its association with nosocomial infections worldwide [1]. In many different bacterial species, virulence is close related to motility [2]. This is the case in *A. baumannii* [3], in which two types of motility have been described: (i) twitching motility, a coordinated multicellular movement driven by the extension, attachment and retraction of type IV pili, and (ii) surface-associated motility, an appendage-independent form of movement [4]. Despite direct connection between twitching motility in virulence has not been observed for *A. baumannii* [2], several reports confirm the importance of surface-associated motility in the virulence of *A. baumannii* [3,5–7]. This type of motility has been associated to the synthesis of 1,3-diaminopropane [5], quorum sensing [8], blue light [9], lipooligosaccharide production [10], and at least to five of the six known efflux pump superfamilies [7].

The motility of bacteria is precisely regulated by a chemotaxis system that enables the cells to sense specific chemicals and then direct movement toward or away from them [11]. A recent report showed that inactivation of the putative chemotaxis CheA/Y-like regulator A1S_2811 in *A. baumannii* strain ATCC 17978 impairs the surface-associated motility of the bacterium [12]. In *Pseudomonas aeruginosa*, another nosocomial pathogen closely related to *Acinetobacter* spp., CheA forms a complex with the chemotactic receptor through the coupling protein CheW. This allows CheA to transfer its phosphate group to CheY, which then interacts with the flagellar motor to switch the direction of flagellar rotation [11]. Although *Acinetobacter* spp. lack flagella, genetic studies in *P. aeruginosa*, which possesses both flagella and pili, have provided insights into a putative pili-mediated chemotaxis pathway, including the homologous components ultimately involved in pili extension/retraction [11].

In *A. baumannii* strain ATCC 17978, homologues to practically all genes encoding putative pili-mediated chemotaxis proteins have been identified in the operon *A1S_2811-2815*, which also includes the gene *A1S*_*2813*, annotated as *pilI*, encoding a CheW-like protein [11,12]. We previously demonstrated that in *Salmonella enterica* the interaction of CheW with RecA, a protein involved in DNA damage repair and recombination, is necessary for swarming motility, a form of flagella-dependent movement across surfaces that involves chemoreceptor cluster formation [13]. In the present work, we show that the direct interaction of RecA and CheW-like proteins is also necessary for the surface-associated motility, chemotaxis and full virulence of *A. baumannii* strain ATCC 17978.

## Materials and methods

### Bacterial strains, plasmids and growth conditions

The bacterial strains and plasmids used in this study are listed in Table 1. *A. baumannii* ATCC 17978 and *Escherichia coli* (strains DH5α, BL21 and OP50) were grown at 37ºC in Luria-Bertani (LB) medium [14] with shaking at 180 rpm. When necessary, the medium was supplemented with kanamycin (50 mg/L), gentamicin (20 mg/L for *E. coli* or 40 mg/L for *A. baumannii*), ampicillin (50 mg/L) or chloramphenicol (34 mg/L). The plasmids pCR-BluntII-TOPO (Invitrogen, catalog number 451245), pVRL1 [15] and pUA1108 [16] were used for mutant construction, complementation and overexpression, respectively (Table 1). The growth of *A. baumannii* was monitored using bacterial cultures in LB broth inoculated with an overnight culture at a dilution of 1:100 and then incubated at 37ºC with shaking at 180 rpm. The optical density at 600 nm (OD_600_) was measured hourly.

**Table 1.** Bacterial strains and plasmids used in this work.

Strains	Relevant characteristics	Source or reference
*Escherichia coli* strains		
DH5α	*E. coli supE4 ΔlacU169 (ϕ80 ΔlacZ ΔM15) hsdR17, recA1, endA1, gyrA96, thi-1, relA1*	Clontech
BL21 (DE3) pLysS	*E. coli* F^−^ *dcm ompT lon hsdS(rB*^−^*mB*^−^*) galλ*(*DE3*) carrying pLysS plasmid, Cm^R^	Stratagene
*Acinetobacter baumannii* strains		
ATCC 17978	Wild-type	ATCC
*recA*	Strain ATCC 17978 with *recA*::Km, Km^R^	[19]
*A1S_2813*	Strain ATCC 17978 with *A1S*_*2813*::pCR-BluntII-TOPO disruption, Km^R^, Zeo^R^	This work
Plasmids		
pCR-BluntII-TOPO	Cloning vector, Km^R^, Zeo^R^	Invitrogen
pVRL1	Complementation vector, Gm^R^	[9]
pUA1108	pGEX 4 T-1 derivative plasmid without the GST fusion tag, carrying the Ptac promoter and the *lacI^q^* gene; used as overexpression vector, Ap^R^	[10]
pUA1108- *recA-FLAG*	pUA1108 plasmid carrying ATCC 17978 *recA* with FLAG tag, Ap^R^	This work
pUA1108-*A1S_2813-6× His*	pUA1108 plasmid carrying ATCC 17978 *A1S_2813* with 6× His tag, Ap^R^	This work
pUA1108-*A1S_2813#S97A-6× His*	pUA1108 plasmid carrying ATCC 17978 *A1S_2813* Ser97Ala with 6× His tag, Ap^R^	This work
pUA1108-*A1S_2813#I121A-6× His*	pUA1108 plasmid carrying ATCC 17978 *A1S_2813* Ile121Ala with 6× His tag, Ap^R^	This work
pVRL1- *recA*	pVRL1 plasmid carrying ATCC 17978 *recA*, Gm^R^	This work
pVRL1-*A1S_2813*	pVRL1 plasmid carrying ATCC 17978 *A1S*_*2813*, Gm^R^	This work
pVRL1-*A1S_2813#S97A*	pVRL1 plasmid carrying ATCC 17978 *A1S_2813* Ser97Ala, Gm^R^	This work
pVRL1-*A1S_2813#I121A*	pVRL1 plasmid carrying ATCC 17978 *A1S_2813* Ile121Ala, Gm^R^	This work

Cm^R^, Km^R^, Zeo^R^, Gm^R^, Ap^R^ stand for resistance to chloramphenicol, kanamycin, zeocin, gentamicin and ampicillin, respectively.

### Accession numbers

The sequences of RecA and CheW-like proteins of *A. baumannii* strain ATCC 17978 used in this study were obtained from NCBI (National Center for Biotechnology Information), with the following accession numbers: APP32573 (RecA; old annotation A1S_1962, current annotation AUO97_17690) and APP29846 (CheW-like protein; old annotation A1S_2813, current annotation AUO97_03055).

### A. baumannii *knockout construction and complementation*

Gene inactivation was carried out as previously described [17]. Briefly, an internal fragment of the target gene from wild-type (WT) *A. baumannii* strain ATCC 17978 was PCR-amplified using the appropriated primers (Supplementary Table 1). The resulting fragment was cloned into the pCR-BluntII-TOPO plasmid (Invitrogen), transformed in *E. coli* DH5α and selected on kanamycin-containing LB plates. The purified plasmid was then introduced into WT *A. baumannii* by electroporation and the resulting transformants were selected on kanamycin-containing plates. Recombinant clones were confirmed by sequencing (Macrogen) of the PCR products obtained by using the appropriate primers, indicated in Supplementary Table 1. Ten passages without selective pressure and colony resuspension were carried out every 24 h to determine the stability of the knockout, by colony counting on LB plates with and without kanamycin at the last passage. For complementation, the target gene was cloned into the pVRL1 vector using the appropriate primers (Supplementary Table 1), which included *Xho*I-*Nco*I restriction sequences [15]. The recombinant plasmid was introduced by electroporation, firstly into *E. col* DH5α and then, once the correct construct had been verified by PCR and sequencing (Macrogen), into the corresponding *A. baumannii* knockout. The complemented mutants were selected on kanamycin- and gentamicin-containing plates.

### Surface-associated motility assays

The assays were conducted on modified fresh LB agar plates (1% tryptone, 0.5% yeast extract, 0.5% NaCl, 0.5% glucose, and 0.5% Difco agar), prepared on the day of the experiment. Bacterial cultures were grown to early stationary phase and a 5-µl droplet of the culture was placed in the center of the plate. The inoculated plates were incubated at 30°C for 16–20 h until the WT strain had grown to reach the plate border. All assays were carried out at least three times in independent experiments, each in triplicate. A ChemiDoc^TM^ XRS+ system (Bio-Rad) was used to obtain representative images.

### Chemotaxis assays

*A. baumannii* movement toward a chemoattractant was assayed as previously described [18], with slight modifications. Briefly, autoclaved, 3-cm-long capillary tubes (Microcaps, Drummond Scientific Co., catalog number 1-000-0010) were half-filled with chemotaxis buffer (CB; 10 mM potassium phosphate, 0.1 mM EDTA, 1 mM MgSO4) solidified using agarose (3% w/v) with or without 2% methanol as chemoattractant and then completely filled with CB without agarose. Bacterial suspensions were prepared from cultures grown to mid-exponential phase (OD_600_ of 0.5) in Tryptone broth (1% tryptone and 0.5% NaCl) and then washed and resuspended in CB to an OD_600_ of 0.1. Chemotaxis chambers were prepared in a six-well plates (Sardstedt), with one plate for each bacterial suspension. A capillary containing either the chemoattractant or CB alone was placed in each well so that it soaked in 2 ml of the appropriate bacterial suspension. After a 2-h incubation in the chemotactic chambers at 30ºC, the capillaries were washed twice to remove any externally attached cells, broken open and their contents poured into capillary tubes containing saline solution. Serial dilutions of the suspensions were plated in LB medium to determine the number of CFU (colony-forming units) per ml obtained under each condition and then normalized between capillaries with and without chemoattractant. The chemotaxis experiments were carried out in at least 9 independent experiments.

### Transmission electron microscopy (TEM)

Colonies of each strain from the motility plates were gently resuspended in 50 µl of 0.1 M phosphate buffer (PB). A 10-μl aliquot of each one was deposited onto carbon-coated film meshes supported by standard copper TEM grids and incubated for 30 min at room temperature. The samples were then fixed for 10 min in a glutaraldehyde solution (1% final concentration) prepared in PB, washed twice with PB and dried. Thereafter, they were negatively stained with 2% uranyl acetate for 1 min and observed on a JEM-1400 transmission electron microscope (JEOL Inc., Peabody, MA, USA).

### Nematode fertility assays

The virulence of *A. baumannii* strains was determined based on a *Caenorhabditis elegans* nematode progeny count assay, performed as previously described [19]. Briefly, the low-virulence strain *E. coli* OP50, grown as a lawn on NGM (Nematode Growth Medium) plates, was used to feed *C. elegans* strain N2. Physiological synchronization was achieved by hatching the nematode eggs in M9 medium (0.02 M KH_2_PO_4_, 0.04 M Na_2_HPO_4_, 0.08 M NaCl, and 0.001 M MgSO_4_) and then arresting the growth of the worms in the first larval (L1) stage overnight at 16°C. The L1 larvae were then cultivated to the last larval stage (L4) on NGM plates seeded with the appropriate bacterial strain. Each L4 worm was placed on a PGS (peptone glucose sorbitol) plate with the corresponding *A. baumannii* strain and incubated at 25°C. During each of the following 3 days, the worms were transferred to new plates seeded with the same bacterial strain. Nematode fertility was determined using a stereomicroscope (Olympus SZ51) to count the progeny daily beginning 48 h after the removal of the parent worm. Six independent replicates were established for each strain and each fertility assay was performed in triplicate.

### Galleria *killing assays*

The virulence of *A. baumannii* strains was also determined using a modified version of the *Galleria mellonella* (wax moth) model described by Peleg *et al* [20]. Briefly, ten caterpillars, each with an approximate weight of 300 mg, were inoculated via the hemocoel with 10 μl of an exponentially growing (OD_600_ = 0.5) strain of *A. baumannii*, previously resuspended in phosphate-buffered saline (PBS). The concentration of the inoculum was confirmed by colony counts on LB agar. As a negative control, 10 μl of PBS was injected into the same number of caterpillars. The caterpillars were incubated at 37°C in dark, and their survival was checked every 12 h for a total of 96 h. All *G. mellonella* killing experiments were performed at least twice. To assess plasmid stability of complemented mutants used in this animal model, at least 5 worms for each strain were infected with sublethal doses of the corresponding complemented mutant (approximately 1 × 10^4^ CFU/worm) and incubated at 37ºC for 96 h. The worms were then washed with ethanol 70% and sterile water. Afterward, the hemolymph was extracted and serial dilutions were plated on solid media containing kanamycin or kanamycin plus gentamycin.‬‬‬‬‬‬‬

‬‬‬‬‬‬‬‬‬‬‬‬‬‬‬‬‬‬‬‬‬‬‬‬‬‬

### Co-immunoprecipitation assays

The *recA* and *A1S_2813* genes were cloned into the pUA1108 overexpression vector after their amplification by PCR using the appropriate primers (Supplementary Table 1). Recombinant plasmids were introduced by electroporation of *E. coli* BL21, selected on ampicillin-containing plates and confirmed by sequencing (Macrogen). Interaction of the RecA and A1S_2813 proteins (carrying FLAG and 6× His tag sequences, respectively) was determined as described by Mayola *et al* [16]., with slight modifications. Briefly, pellets obtained from IPTG-induced cultures were sonicated and the supernatants collected after centrifugation. The cell lysates were immuneseparated using Pure Proteome Protein A magnetic beads (Millipore, catalog number LSKMAGA10) coated with the appropriate primary antibodies: rabbit anti-FLAG (Sigma-Aldrich, catalog number F7425) or mouse anti-6× His IgGs (Roche, catalog number 11922416001), according to the manufacturer’s instructions. The samples were separated by SDS-PAGE on a 15% polyacrylamide gel and analyzed by western blotting. Horseradish peroxidase (HRP)-coupled goat anti-rabbit (Yo proteins, catalog number 464) or anti-mouse IgGs antibodies (Acris, catalog number R1253HRP) were used as secondary antibodies and the immune complexes were visualized by the addition of the Luminata ForteTM western HRP substrate (Millipore, catalog number WBLUF0100). Representative membrane images were obtained using a ChemiDocTM XRS+ system (Bio-Rad).

### Site-specific mutagenesis of CheW-like protein

The ORF of *A. baumannii* CheW-like protein was cloned into the overexpression plasmid pUA1108 and specific nucleotides were then replaced using the appropriate oligonucleotides (Supplementary Table 1) and the Q5® Site-Directed Mutagenesis kit (BioLabs, catalog number E0554S) according to the manufacturer’s instructions. Sequence sites to mutagenize were selected based on comparisons of the predicted secondary structures of the *A. baumannii* CheW-like and *S. enterica* CheW proteins (accession numbers: ABO13219 and NP_460877, respectively), performed with the SABLE program (http://sable.cchmc.org/). The pUA1108 plasmids containing genes coding either RecA-FLAG or A1S_2813-6× His and the derivate site-specific mutagenic proteins were introduced by electroporation into *E. coli* BL21 (DE3) pLysS (Promega) and selected on ampicillin- and chloramphenicol-containing plates. Additionally, all *A1S_2813* genes carrying a specific mutagenized sequence were cloned from the pUA1108 plasmid into the pVRL1 vector using the appropriate primers containing *Xho*I-*Not*I restriction sequences (Supplementary Table 1) and introduced into the *A. baumannii A1S_2813* mutant strain. The transformants were selected on kanamycin- and gentamicin-containing plates. All constructs were verified by sequencing (Macrogen).

### Statistical analysis

Data obtained in the chemotaxis assays and the nematode model of virulence are presented as the mean ± standard deviation and were analyzed using a two-tailed, one-way analysis of variance (ANOVA), followed by the Tukey test for *post-hoc* multiple group comparisons. In the *G. mellonella* killing assay, survival curves were plotted using the Kaplan-Meier method and differences in survival were calculated using the log-rank test. In all tests, a P value < 0.05 was considered to indicate statistical significance.

## Results

### *RecA and CheW-like proteins are involved in the surface-associated motility and chemotaxis of* A. baumannii *strain ATCC 17978.*

**Figure 1.**
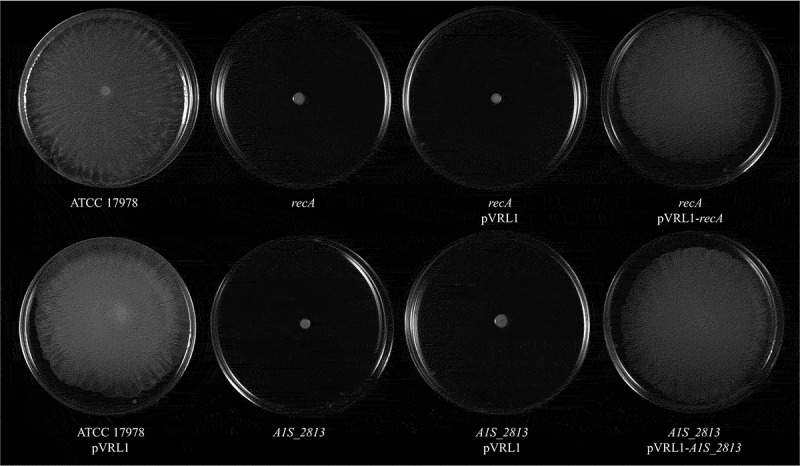
Representative image of the surface-associated motility of the indicated *A. baumannii* strains.

**Figure 2.**
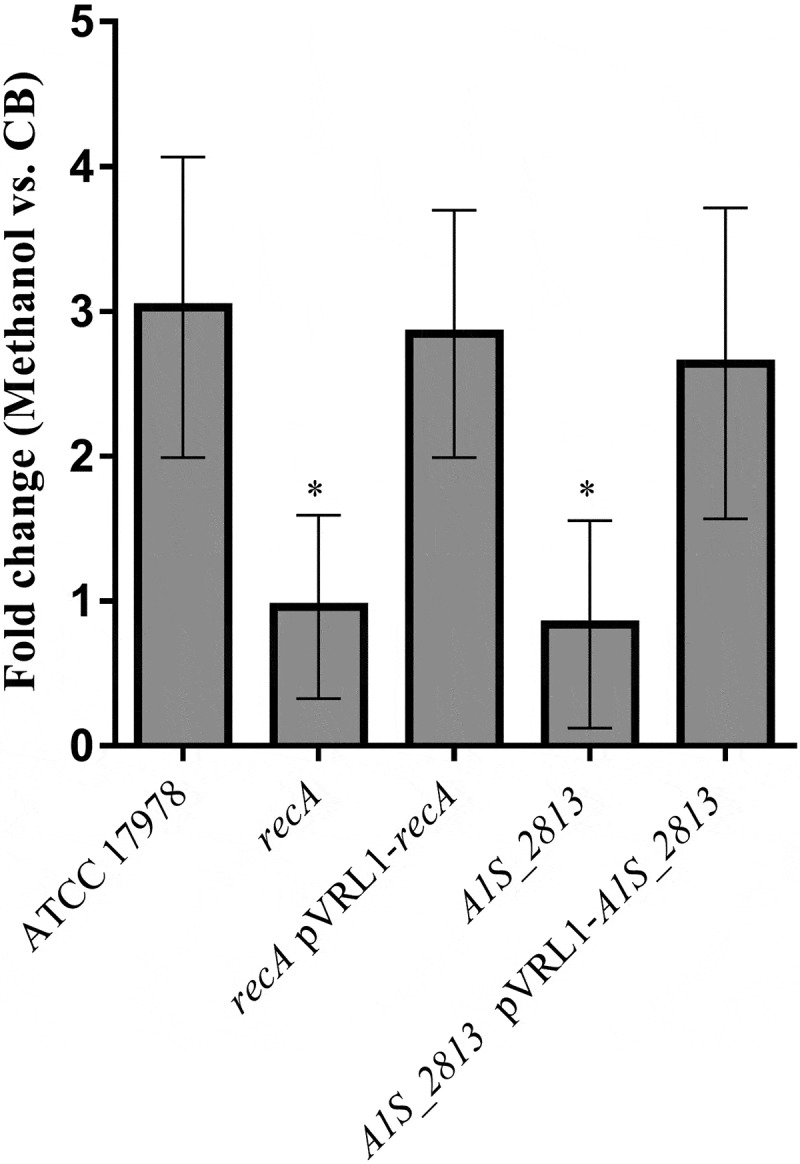
The fold-change between viable bacteria (CFU) of the indicated strains, calculated as the number of CFU in capillaries containing chemoattractant (2% methanol) divided by the number in control capillaries containing only CB (chemotaxis buffer). Error bars represent the standard deviation (SD) of the means. *P < 0.05 in a comparison with the *A. baumannii* parental strain (ATCC 17978) and based on at least nine independent experiments.

**Figure 3.**
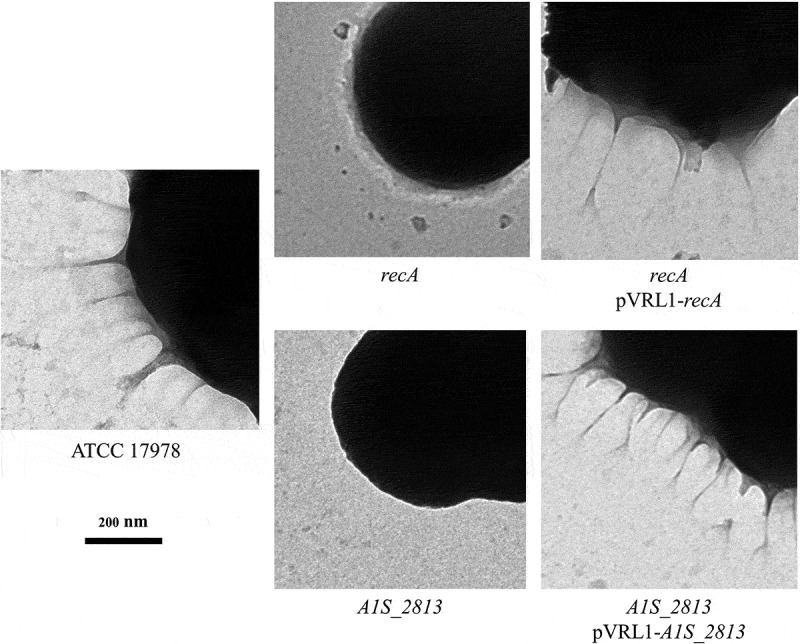
Transmission electron microscopy of the indicated *A. baumannii* strains. The images were obtained at 4,000 × magnification.

After 24 h of incubation on motility plates, the *recA* and the *A1S_2813 A. baumannii* knock-out mutants (Table 1) did not show surface-associated motility, unlike the WT parental strain *A. baumannii* ATCC 17978 (Figure 1). To link the impaired motility to the lack of either the *recA* or the *A1S_2813* gene, the respective WT genes were introduced into the corresponding knockout-mutant strains. The resulting complementation restored surface-associated motility by both mutants to a level similar to that observed in the WT strain (Figure 1). By contrast, introduction of the empty expression vector pVRL1 did not modify the behavior of any of the studied strains (Figure 1). Since the growth curves of the *recA* and *A1S_2813* knockout mutants (both presenting a stability of >90%) cultured in LB medium were comparable to that of the WT strain (Fig. S1), the altered surface-associated motility of the mutants cannot be attributed to reductions in the bacterial growth rate. To determine whether the lack of RecA and/or CheW-like proteins affects bacterial chemotaxis, a fishing method was used in which methanol served as the chemoattractant. The results showed an approximately 3-fold increase in bacterial counts of the WT strain in capillaries with methanol compared to the counts obtained in capillaries containing only CB (control), whereas in the case of both the *recA* and *A1S_2813* knockout mutants there were no significant differences of theses mutants in cell migration toward methanol compared to the control capillaries containing only CB, showing a significant difference in the fold-change cell count in both mutants with respect to that observed in the WT parental strain (Figure 2). Furthermore, the complementation of either mutant restored the WT chemotaxis phenotype (Figure 2). In addition, on the TEM images, both *recA* and *A1S_2813* knockouts lacked the pilus‐like structure typically present on cells of the ATCC 17978 WT strain, but in each mutant complementation resulted in its recovery (Figure 3 and Fig. S2).

### *The lack of either RecA or cheW-like proteins reduces the virulence of* A. baumannii *strain ATCC 17978 in two different animal models.*

**Figure 4.**
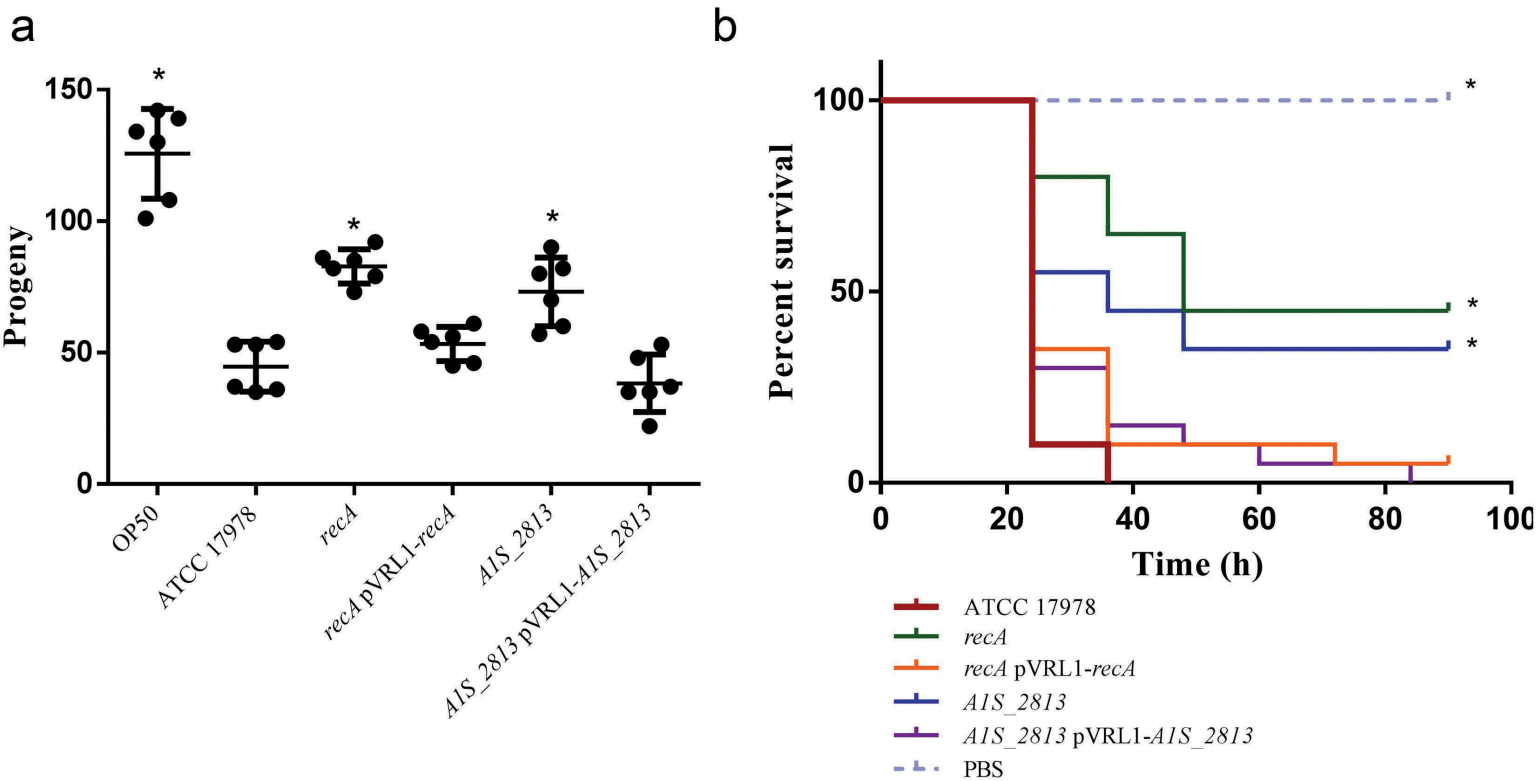
(a) Representative results of a *C. elegans* fertility assay using the indicated strains of *A. baumannii*. *P < 0.05 compared to the parental ATCC 17978 strain. *E. coli* strain OP50 was included as a low virulent control. (b) Representative *G. mellonella* killing assay of the specified strains. Larvae (n = 10 per group) were inoculated with either ~10^6^ CFU of the indicated strain or PBS (as a negative control). Error bars represent the SDs of the means. *P < 0.05 in a comparison with the *A. baumannii* parental strain (ATCC 17978).

In the *C. elegans* fertility assay, worms fed either the *recA* or the *A1S_2813* mutant exhibited an increased number of progeny compared to worms fed the WT strain (Figure 4(a)), indicating a significant reduction in the pathogenicity of the mutants. When the worms were fed the complemented strains (presenting a plasmid stability of 109 ± 28% and 94 ± 10% for the complemented *recA* and *A1S_2813* mutants, respectively), the number of progeny was less than obtained with the respective mutants and similar to that obtained when the WT strain was used as nourishment (Figure 4(a)). Accordingly, in the *G. mellonella* killing assay, approximately 50% of the worms inoculated with either the *recA* or the *A1S_2813* mutant survived during the 96-h experiment, whereas practically all larvae inoculated with the WT or the complemented strains died within the first 40 h (Figure 4(b)).

### In vitro *interaction between the* A. baumannii *strain ATCC 17978 RecA-FLAG and CheW-like-6× His protein*

**Figure 5.**
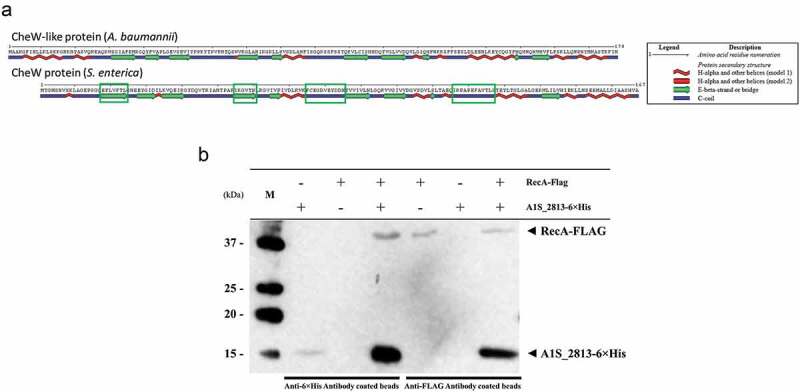
(a) Predicted secondary structure of the *S. enterica* CheW and *A. baumannii* CheW-like proteins. The green boxes indicate the *S. enterica* CheW domains involved in the interaction with RecA. (b) Results of a co-immunoprecipitation assay between the *A. baumannii* RecA-FLAG and A1S_2813-6× His proteins. The supernatants were separated by SDS-PAGE and assessed by western blotting. The image is representative of three independent experiments using three different lysates for each protein. The presence (+) or absence (−) of the RecA-FLAG or A1S_2813-6× His proteins in the corresponding lysate mixture is indicated, as is that of the antibody-coated beads used in each mixture. The RecA-FLAG and A1S_2813-6× His bands detected on a western blot are shown. M: molecular mass marker.

*In silico* analysis revealed that while *S. enterica* CheW and the *A. baumannii* CheW-like protein had an identity of only 24%, their secondary structures were highly similar (Figure 5(a)). In *S. enterica*, the RecA and CheW proteins interact [16]. To determine whether this was also the case for the RecA and CheW-like (A1S_2813) proteins of *A. baumannii*, we conducted an *in silico* modeling of interaction (Fig. S3) and a co-immunoprecipitation assay using the overexpressed tagged proteins RecA-FLAG and A1S_2813-6× His (Figure 5(b)). When the tagged proteins were mixed with their corresponding antibody-coated beads (RecA-FLAG with anti-FLAG or A1S_2813-6× His with anti-6× His), the matching protein was detected in the recovered supernatant (Figure 5(b)). When the tagged proteins were mixed with the opposite antibody-coated beads (RecA-FLAG with anti-6× His or A1S_2813-6× His with anti-FLAG), no proteins were detected. These results confirmed the specificity of the assay (Figure 5(b)). We then added a mixture of the tagged proteins (RecA-FLAG and A1S_2813-6× His) to each suspension of antibody-coated beads, which resulted in the recovery of the opposite tagged protein (RecA-FLAG with anti-6× His and A1S_2813-6× His with anti-FLAG) and its subsequent detection in a western blot assay (Figure 5(b)). Together, these findings clearly demonstrated that RecA-FLAG and the CheW-like-6× His protein of *A. baumannii* specifically interact *in vitro*.

**Figure 6.**
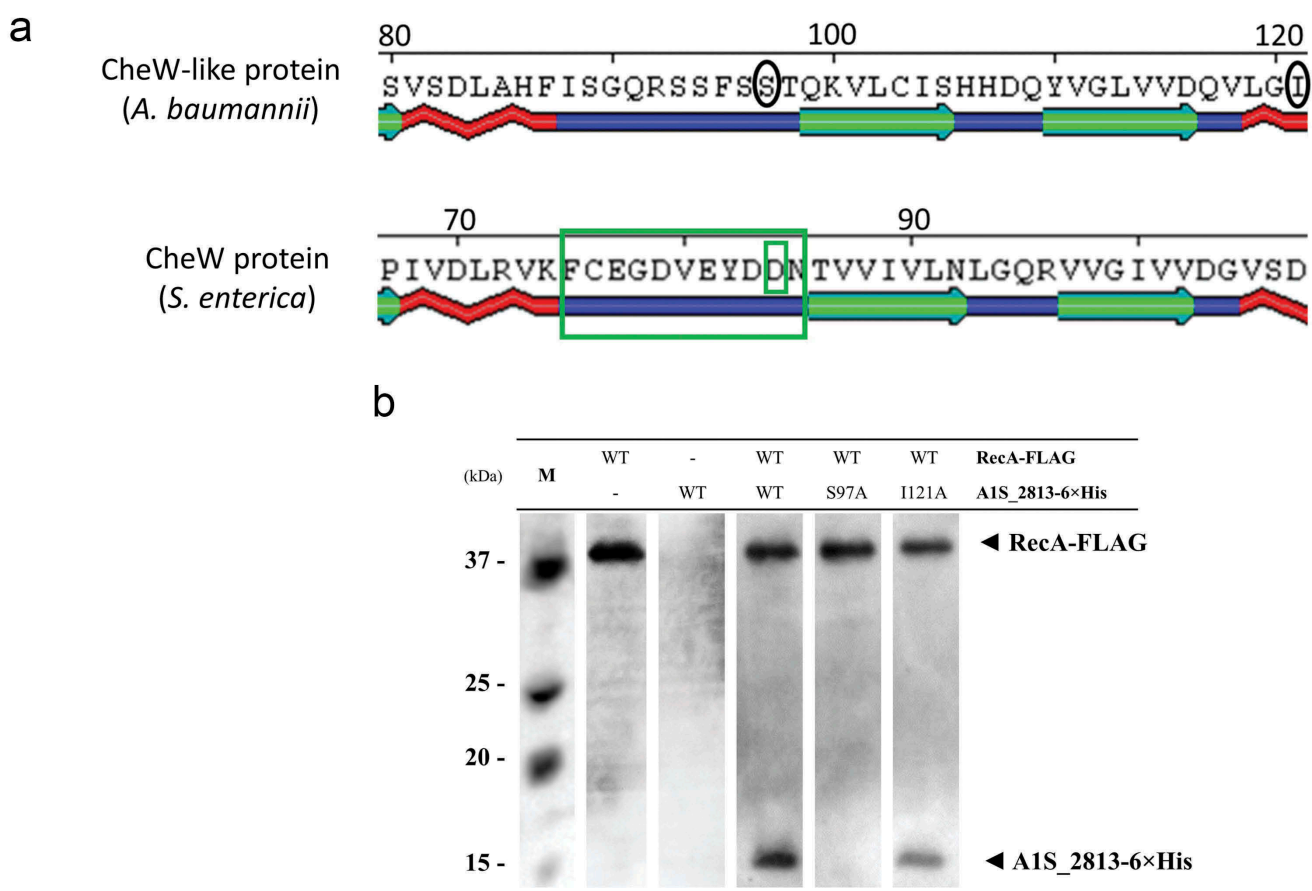
(a) Detail of a selected conserved region in the predicted secondary structure of the *S. enterica* CheW and *A. baumannii* CheW-like proteins. The large and small green boxes indicate, respectively, a known domain and a residue of *S. enterica* CheW protein involved in the interaction with RecA. The black circles indicate the subsequently mutagenized residues from the *A. baumannii* CheW-like protein. (b) Co-immunoprecipitation of the RecA-FLAG and A1S_2813-6× His proteins and the derivative A1S_2813-6× His site-specific mutagenized proteins. The supernatants were separated by SDS-PAGE and assessed by western blotting. The images are representative of those from three independent assays. The presence of the RecA-FLAG and A1S_2813-6× His proteins (WT) and the absence of (-) or residue change (Ser97Ala or Ile121Ala) in A1S_2813-6× His in the corresponding mixtures are indicated. The western blot shows the RecA-FLAG and A1S_2813-6× His protein bands revealed following incubation of the lysates with anti-FLAG coated beads. M: molecular mass marker.

To examine whether the CheW and RecA interaction domains in *S. enterica* are conserved in *A. baumannii*, a CheW-like-6× His derivate-tagged protein was generated by the substitution of residue Ser97Ala. This position of the CheW-like-6× His protein of *A. baumannii* corresponds exactly to the Asp that is the second-to-last residue of the Turn-6 region of *S. enterica* CheW (Figure 6(a)). In CheW, replacement of the Asp by an alanine, a non-reactive amino-acid residue [21], abolishes its interaction with RecA [13]. As a negative control, we also generated, a CheW-like-6× His derivate protein containing the substitution Ile121Ala at a site corresponding to a region of the *S. enterica* CheW protein that is not involved in the interaction with RecA (Figure 6(a)). It is worth noting that appropriate expression of all proteins used in this work was confirmed through western blot assay (Fig. S4). Co-immunoprecipitation assays showed that the RecA-FLAG protein was not able to bind the CheW-like-6× His protein containing the substitution Ser97Ala, whereas the derivate Ile121Ala was recovered (Figure 6(b)). These results indicate that in the CheW-like-6× His protein Ser97 but not Ile121 is essential for the interaction with RecA in *A. baumannii*. Thus, at least one of the essential domains involved in the interaction of CheW with RecA in *S. enterica* is also required for the interaction of the corresponding homologues in *A. baumannii*.

### In vivo *interaction between the* A. baumannii *ATCC 17978 RecA and CheW-like protein*

**Figure 7.**
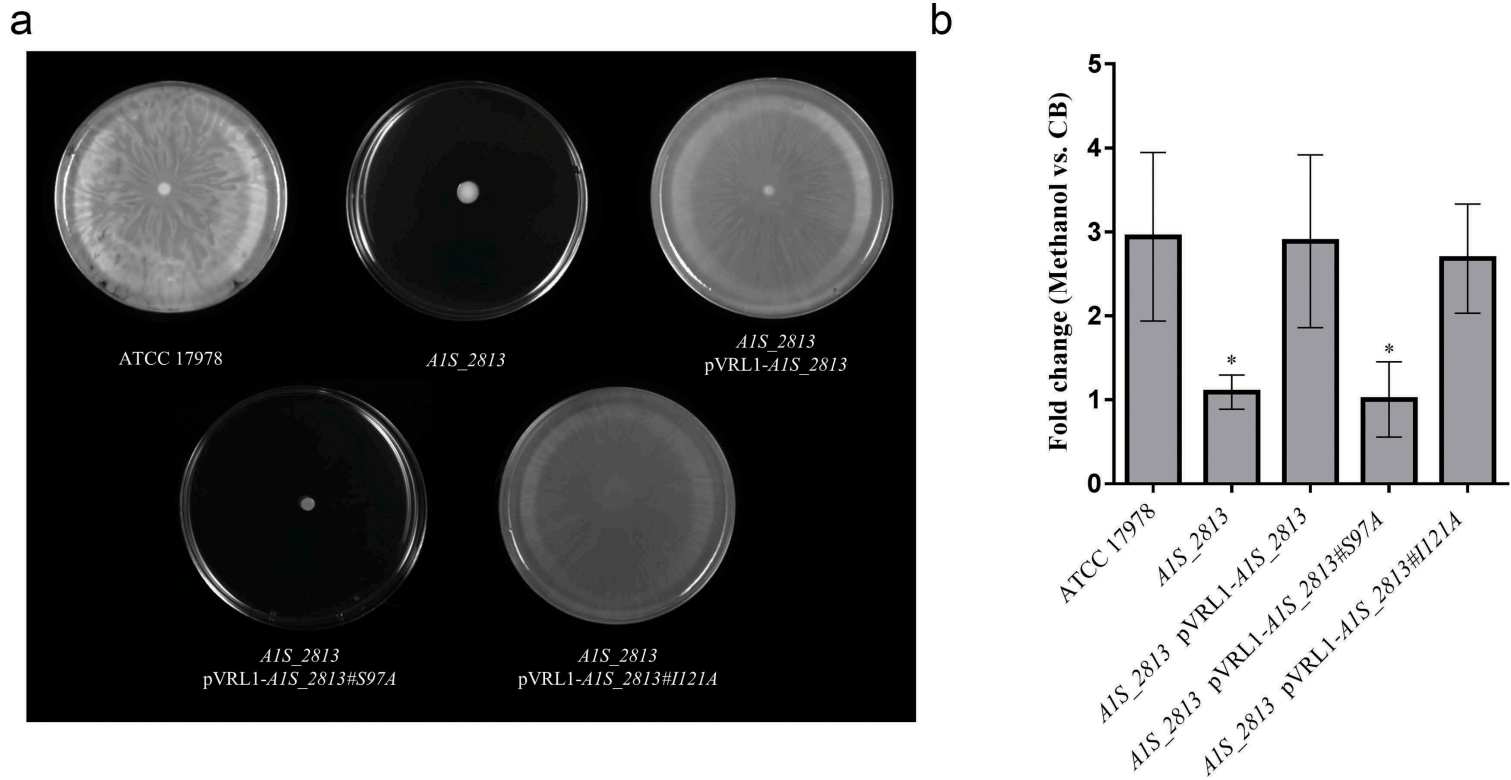
(a) Representative image of the surface-associated motility of the indicated *A. baumannii* strains. (b) The fold-change in the number of viable bacteria (CFU) of the indicated strains, based on the counts in capillaries containing chemoattractant (2% methanol) divided by those in control capillaries containing only CB. Error bars represent the SDs of the means. *P < 0.05 in a comparison with the *A. baumannii* parental strain (ATCC 17978) as determined in at least nine independent experiments.

**Figure 8.**
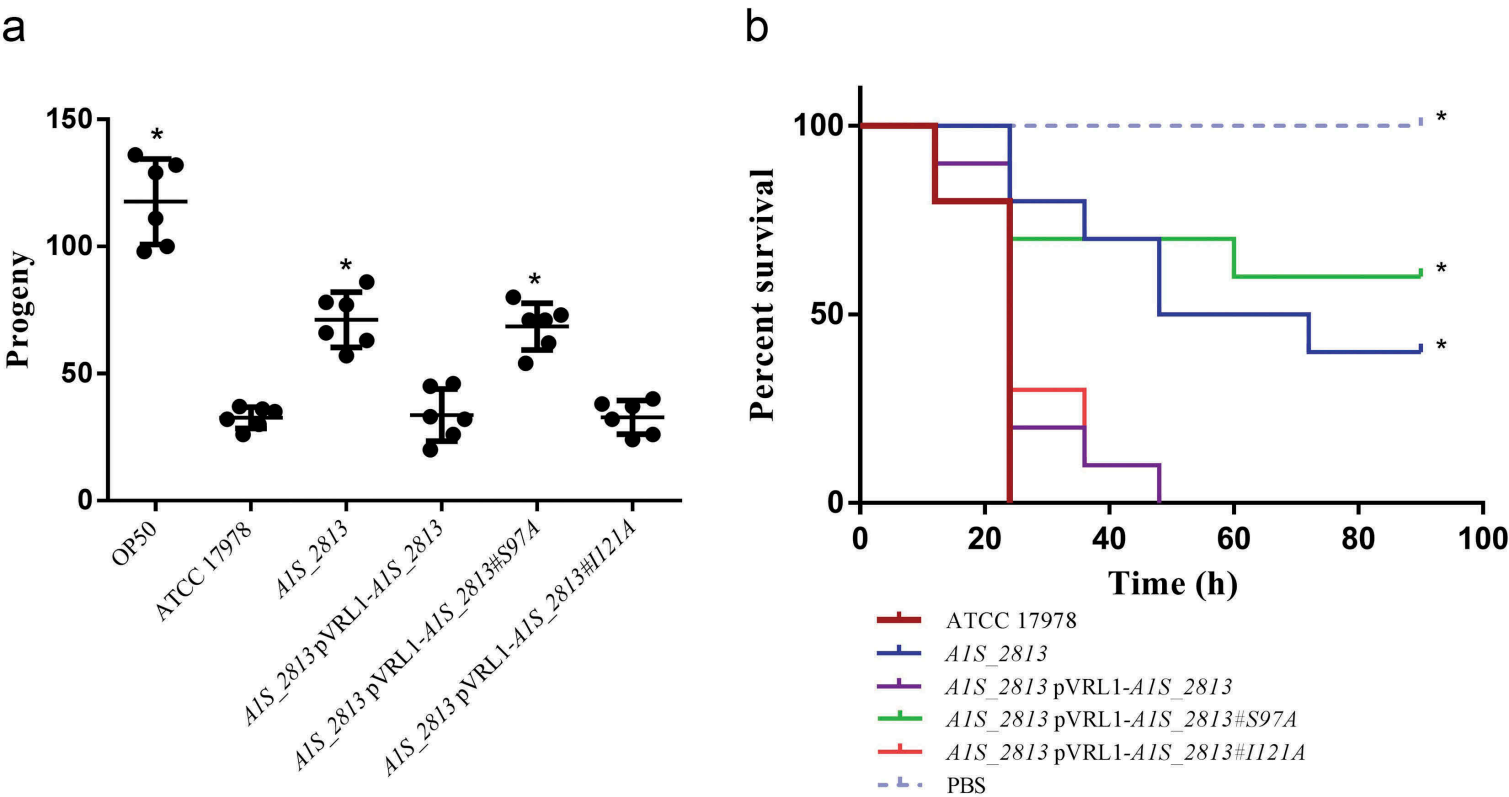
(a) Representative results of a *C. elegans* fertility assay with the indicated strains. *P < 0.05 in a comparison with the *A. baumannii* parental strain (ATCC 17,978). *E. coli* strain OP50 was included as a low virulent control. (b) Representative results of a *G. mellonella* killing assay of the specified strains. Larvae (n = 10 per group) were inoculated with ~10^6^ CFU of the indicated strain or PBS (as a negative control). Error bars represent the SDs of the means. *P < 0.05 in a comparison with the *A. baumannii* parental strain (ATCC 17978).

To determine whether the RecA-A1S_2813 interaction is involved in surface-associated motility and virulence, mutant *A1S_2813* genes encoding the proteins that included the Ser97Ala or Ile121Ala mutations were cloned into the pVRL1 plasmid and introduced into the *A1S_2813* mutant strain. Motility and chemotaxis assays showed that expression in the *A1S_2813* knockout of the mutagenized *A1S_2813* gene whose product included the Ile121Ala substitution completely restored the parental phenotypes (Figure 7). By contrast, the *A1S_2813* knockout expressing the A1S_2813 protein containing the Ser97Ala mutation and thus unable to interact with RecA *in vitro*, did not restore either surface-associated motility or chemotaxis (Figure 7). Finally, the pathogenicity of both strains was tested in the *C. elegans* fertility and *G. mellonella* killing assays (Figure 8). As expected, the virulence of the *cheW*-like mutant expressing the *A1S_2813* gene encoding Ser97Ala mutant protein was impaired in both animal models and was similar to the *A1S_2813* knockout strain (Figure 8). However, the virulence of the *A1S_2813* mutant strain was fully restored by expression of the gene encoding a CheW-like protein containing the Ile121Ala substitution, as occurred when the *A1S_2813* strain was complemented with the corresponding WT gene (Figure 8). These results demonstrated that the interaction between the RecA and CheW-like proteins *in vivo* is necessary for the surface-associated motility and full virulence of *A. baumannii*.

## Discussion

The surface-associated motility characteristic of several *A. baumannii* clinical isolates allows their movement over wet surfaces independent of the bacterial expression of type IV pili [2]. In *A. baumannii*, a non-flagellated bacteria, the molecular and genetic basis of surface-associated motility is unclear and the terminology used in the literature to describe it is often confusing. For example, in several publications surface-associated motility is referred to as swarming, which is, however, flagella-dependent. The surface-associated motility of *A. baumannii* seems to be closely related to the pathogenicity of the bacterium, as an increase in motility is associated with an increase in virulence [6] whereas *A. baumannii* mutants unable to conduct surface-associated motility exhibit an attenuated virulence [3,7].

The motility phenotypes in *A. baumannii* are diverse and include motility on semi-solid media (surface-associated motility), migration conducted by type IV pili, such as occurs at the medium-plastic interface of solid medium (twitching motility), both motilities or neither [22]. Strain ATCC 17978, one of the most well studied clinical isolates of *A. baumannii*, is capable of surface-associated motility but not twitching [22]. In this strain, surface-associated motility was recently linked to the CheA/Y-like protein A1S_2811, a component of a hypothetical chemotactic signal transduction system whose inactivation causes a significant decrease in this type of movement [12]. Our group recently reported that in *S. enterica* the interaction between the chemotaxis system and the RecA protein is necessary for the swarming motility of this species [13]. Specifically, RecA interacts with CheW to allow chemoreceptor cluster formation, which is ultimately involved in flagellar rotation [13]. In previous work, we constructed an *A. baumannii* mutant lacking RecA, the major enzyme involved in recombination, to show the protein’s involvement in virulence as well [23]. The results of the present study demonstrate that inactivation of the *A. baumannii recA* gene also impairs motility (Figure 1). Since a putative operon involved in *A. baumannii* chemotaxis has been recently described, we proceeded to inactivate its *A1S_ 2813* gene (annotated as *pilI* in ATCC 17978 strain [12]), which encodes a protein homologous to the CheW-like protein of *P. aeruginosa* [11]. As in the case of the *recA* mutant, the absence of the gene encoding the CheW-like protein of *A. baumannii* resulted in the abolishment of surface-associated motility (Figure 1), indicating that both RecA and the hypothetical chemotaxis operon *A1S_2811-2815* are involved in the surface-associated motility of *A. baumannii* strain ATCC 17978.

The *A1S_2811* gene (encoding a CheA/Y-like protein) and the *A1S_2813* gene (encoding a CheW-like protein) belong to the same hypothetical chemotactic operon. However, while the importance of this operon in surface-associated motility of *A. baumannii* has been unequivocally demonstrated, to our knowledge, its role in the chemotaxis of this bacterium was previously unknown. The present work is the first to demonstrate that both the CheW-like protein and RecA are involved in the chemotaxis of *A. baumannii* (Figure 2). The molecular machinery that controls chemotaxis in bacteria has been grouped in three different functional classes: flagella, type IV pili, and alternative cellular functions, including those that are acquired by horizontal gene transfer or duplication [24]. For instance, *Myxococcus xanthus*, a gram-negative soil bacterium with a complex life cycle, lacks flagella but makes use of two other types of motility, one dependent and the other independent of type IV pili. It also has at least eight chemosensory systems, most of which play various roles in controlling either one or the other type of motility [25]. Furthermore, *M. xanthus* possesses a CheW homologue that is required for the biogenesis of the cell surface appendages necessary for type-IV-pili-independent motility [26]. By contrast, in *A. baumannii* strain ATCC 17978 only one chemosensory system (the *A1S_2811-2815* operon) has been identified so far. It should be implicated in the chemotactic response through the regulation of appendages that are distinct from flagella and type IV pili and/or through appendage-independent mechanisms.

In *P. aeruginosa*, a pili-mediated chemotaxis pathway has been described in which the CheW-like protein is the coupling protein between the chemotactic receptor and the CheA/Y-like two component system. The loss of the CheW-like protein of *A. baumannii* strain ATCC 17978 reduces surface-associated motility as well as the number of pilus‐like structures and the amount of cell-surface extrapolymeric substances [11,12]. The present study similarly showed a dramatic reduction of both pilus-like structures and secreted substances in the *recA* and *cheW*-like mutant derivatives of *A. baumannii* strain ATCC 17978 (Figure 3). This finding is consistent with our previous work, in which we showed that the inactivation of transporters belonging to practically all families of *A. baumannii* efflux pumps drastically reduces the surface-associated motility of the bacterium [7]. The reduction might be due to the decreased translocation of molecules related to this type of motility, such as surfactants or quorum-sensing signals [5,8,10]. Accordingly, one of the most strongly down-regulated genes of an *A. baumannii cheA/Y*-like mutant belongs to the operon *A1S_0112-0119*, involved in the production of acinetin 505 and its extrusion via an RND efflux pump [12,27]. Acinetin 505 is a lipopeptide that may act as a surfactant in the promotion of surface-associated motility [27].

The impact of CheW-like and RecA proteins on the virulence of *A. baumannii* was tested in two different animal models, based on *C. elegans* and *G. mellonella*. Both showed the significantly decreased virulence of the *recA* and *cheW*-like knockout mutants (Figure 4). The results were in agreement with those obtained using a RecA mutant in a mouse model [23]. Together, these studies support a clear relationship between the surface-associated motility and the pathogenesis of *A. baumannii*. Remarkably, all ATCC 17978 mutant derivatives analyzed so far presenting a decreased surface-associated motility also show a reduction in their virulence [3,7]. On the other hand, the *A. baumannii* strain lacking the transcriptional repressor H-NS is not only hypermotile but also shows an increased virulence [6]. In both types of mutants, efflux pumps are involved in the observed phenotype, albeit in an opposite manner. In mutants lacking surface-associated motility, the expression of transporters involved in the extrusion of components relevant to this type of motility are absent or reduced [3,7]. In the hypermotile mutant, the expression of genes encoding quorum-sensing signals, a novel type I pilus and several efflux pumps, including those of the aforementioned *A1S_0112-0119* operon, is significatively increased [6].

In flagella-bearing *S. enterica*, the interaction of its RecA and CheW-like homologues is necessary for chemotaxis and swarming motility [13]. We therefore asked whether a similar interaction was required in *A. baumannii* ATCC 17978, in which the two proteins were shown to be necessary for surface-associated motility, chemotaxis and virulence. Co-immunoprecipitation assays using the *A. baumannii* RecA and CheW-like proteins revealed the highly conserved secondary structure of the latter with respect to its *S. enterica* homologue CheW (Figure 5(a)) and the specific interaction of *A. baumannii* RecA and CheW-like proteins (Figure 5(b)). In addition, interaction of the CheW-like protein with *A. baumannii* RecA was abolished by the introduction of a mutation into one of the former’s protein domains that is structurally conserved and whose homologue in *S. enterica* is involved in the RecA-CheW interaction (Figure 6(a,b)). The mutation that abolished this interaction was the same that led to impaired surface-associated motility, chemotaxis and virulence (Figures 7 and 8), which clearly demonstrated that the interaction between RecA and CheW-like proteins is necessary for these functions in *A. baumannii* strain ATCC 17978.

In *S. enterica* the interaction between RecA and CheW plays a key role in swarming motility [13]. Specifically, the adaptor CheW couples the chemoreceptor protein to CheA, a histidine kinase that transfers the signal to the CheY response regulator, which acts on the flagellar motor by switching flagellar rotation [28]. RecA contributes to altering the distribution of CheW at the cell poles, at sites where flagella are distributed, to modulate swarming motility [13]. In *P. aeruginosa*, a pili-mediated chemotaxis pathway has been proposed that contains homologous components ultimately involved in pili extension/retraction [11]. Homologies of most of these proteins are also present in *A. baumannii* strain ATCC 17978, including a CheA/Y-like protein involved in surface-associated motility [12] and, as demonstrated herein, the CheW-like protein. Thus far, a role for RecA in chemotaxis and motility has been reported only in *S. enterica*, as described above; however, our results evidence the importance of the protein in the surface-associated motility of *A. baumannii* strain ATCC 17978. Since the latter bacterium lacks flagella and mutants deficient in functional type IV pili are still able to conduct surface-associated motility [29], then the interaction between the *A. baumannii* RecA and CheW-like proteins must control appendages different from either flagella or type IV pili. Alternatively, or in addition, the two proteins may control the expression of genes related to surface-associated motility, perhaps through a non-traditional chemosensory system such as that described above for *M. xanthus* [25].

In summary, our study reveals a novel role for the RecA protein in *A. baumannii* strain ATCC 17978 and demonstrates that its interaction with the CheW-like protein of the chemotactic system is necessary for surface-associated motility, chemotaxis and the full virulence of the bacterium.

## Supplementary material

The supplementary data for this article can be accessed here.
