# Supplementary material for: Direct interaction between RecA and a CheW-like protein is required for surface-associated motility, chemotaxis and the full virulence of Acinetobacter baumannii strain ATCC 17978
Source: Virulence. 2020 Apr 7;11(1):315–26. doi: 10.1080/21505594.2020.1748923 (PMC7161683; doi:10.1080/21505594.2020.1748923)
Supplement: Supplemental Material [file kvir-11-01-1748923-s001.zip › SUPPLEMENTARY FIGURE LEGENDS.docx]

**SUPPLEMENTARY FIGURE LEGENDS**

**Figure S1.** Growth curves of the indicated *A. baumannii* strains.

**Figure S2.** Transmission electron microscopy of the indicated *A. baumannii* strains obtained as described above but without the use of glutaraldehyde. The images were obtained at 4,000 × magnification.

**Figure S3.** *In silico* model for the interaction of the *A. baumannii* RecA and CheW-like protein analyzed using PyMOL software as previously described^13^.

**Figure S4.** Western blot of the indicated lysates using the appropriate antibodies. C-: negative control. M: molecular mass marker.
