# Supplementary material for: Direct interaction between RecA and a CheW-like protein is required for surface-associated motility, chemotaxis and the full virulence of Acinetobacter baumannii strain ATCC 17978
Source: Virulence. 2020 Apr 7;11(1):315–26. doi: 10.1080/21505594.2020.1748923 (PMC7161683; doi:10.1080/21505594.2020.1748923)
Supplement: Supplemental Material [file kvir-11-01-1748923-s001.zip › Supplementary Table 1.docx]

**Supplementary Table 1.** Oligonucleotides used in this work.

| **Name** | **Sequence (5’ to 3’)** | **Application** |
| --- | --- | --- |
| A1S_2813IntFw | GATTGGCAAACATTC | Mutant construction |
| A1S_2813IntRv | CACATACTGGTCATGATG | Mutant construction |
| RecAComplFw | CAGTCTCGAGATGGATGAGAATAAAAGC | Complementation |
| RecAComplRv | CAGTCCATGGTTACGATTCTAATAAAAGATC | Complementation |
| A1S_2813ComplFw | CAGTCTCGAGATGGCAGCGAATGGATTTATCG | Complementation and mutant verification |
| A1S_2813ComplRv | CAGTCCATGGTTAGTTTATAAATTTTGTCGA | Complementation and mutant verification |
| RecAFLAGFw | GGAATTCCATATGGATGAGAATAAAAGC | Cloning tagged-protein in pUA1108 |
| RecAFLAGRv | CGGGATCCTTA**TTTGTCGTCGTCGTCTTTGTAGTC**gccgccgccCGATTCTAATAAAAGATC | Cloning tagged-protein in pUA1108 |
| A1S_2813HisFw | GGAATTCCATATGAGAGGGCAATACTTC | Cloning tagged-protein in pUA1108 |
| A1S_2813HisRv | CGGGATCCTTA**ATGATGATGATGATGATG**gccgccgccGTTTATAAATTTTGTCGA | Cloning tagged-protein in pUA1108 |
| A1S_2813S97AFw | TTCATTTTCGGCTACCCAAAAAG | A1S_2813#S97A mutant derivate construction |
| A1S_2813S97ARv | CTCCGCTGCCCTGAAATA | A1S_2813#S97A mutant derivate construction |
| A1S_2813I121AFw | GGTTTTGGGGGCTCAGCACTTTAATAAAAAAAG | A1S_2813#I121A mutant derivate construction |
| A1S_2813I121ARv | TGATCCACAACCAACCCC | A1S_2813#I121A mutant derivate construction |
| -108pUA1108 | CCGACATCATAACGGTTC | Sequencing primer for pUA1108 vector |
| +128pUA1108 | AGACAAGCTGTGACCGTC | Sequencing primer for pUA1108 vector |
| M13FpUC | GTTTTCCCAGTCACGAC | Sequencing primer for pCR-BluntII-TOPO and pVRL1 vectors |
| M13RpUC | CAGGAAACAGCTATGAC | Sequencing primer for pCR-BluntII-TOPO and pVRL1 vectors |

Restriction endonuclease sites are underlined, FLAG and 6×His tags are indicated in bold and Gly spacers in lower case.
