# Supplementary figures and images for: Direct interaction between RecA and a CheW-like protein is required for surface-associated motility, chemotaxis and the full virulence of Acinetobacter baumannii strain ATCC 17978
Source: Virulence. 2020 Apr 7;11(1):315–26. doi: 10.1080/21505594.2020.1748923 (PMC7161683; doi:10.1080/21505594.2020.1748923)

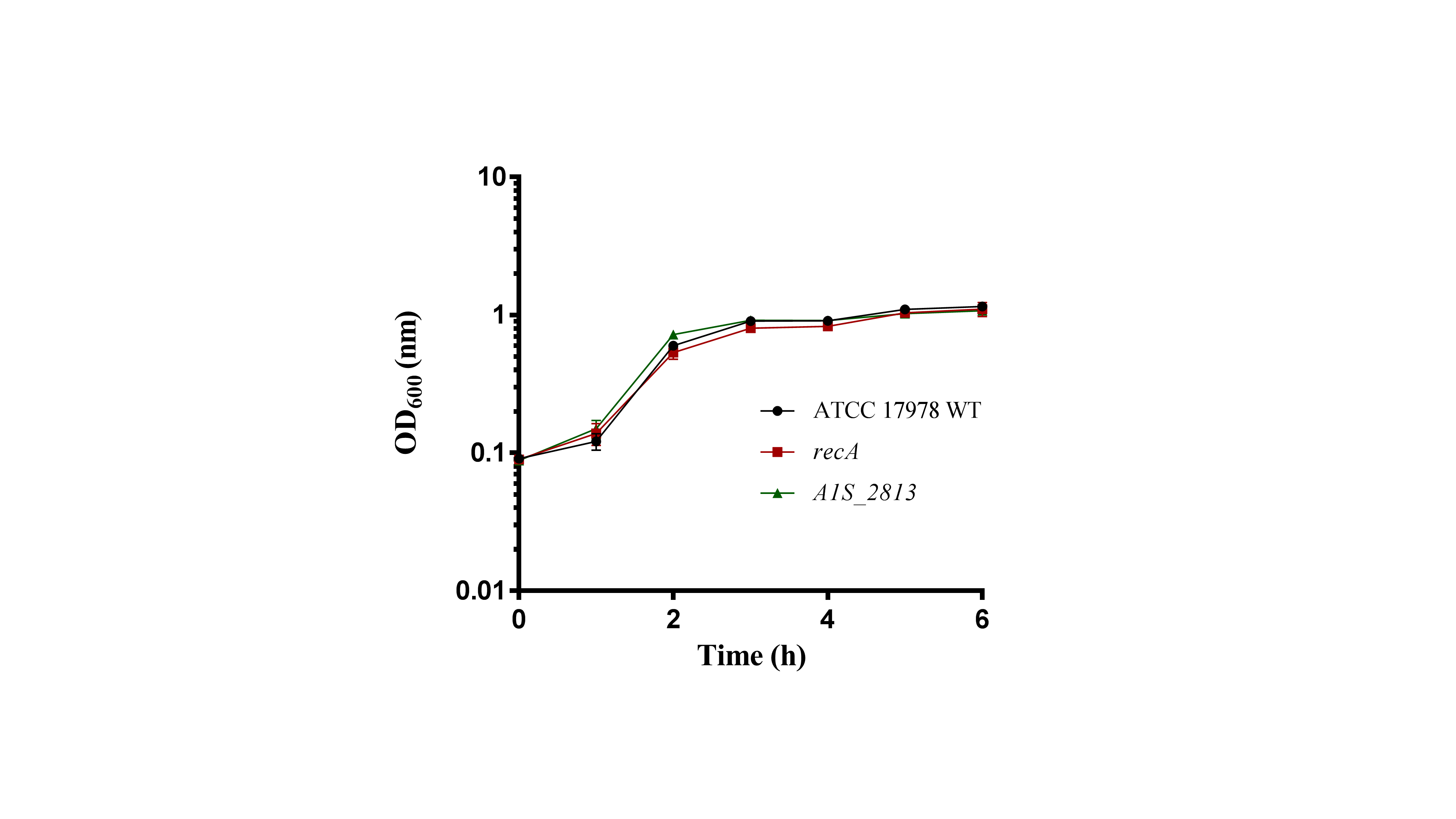

Supplement: Supplemental Material [file kvir-11-01-1748923-s001.zip › Fig. S1.TIF]

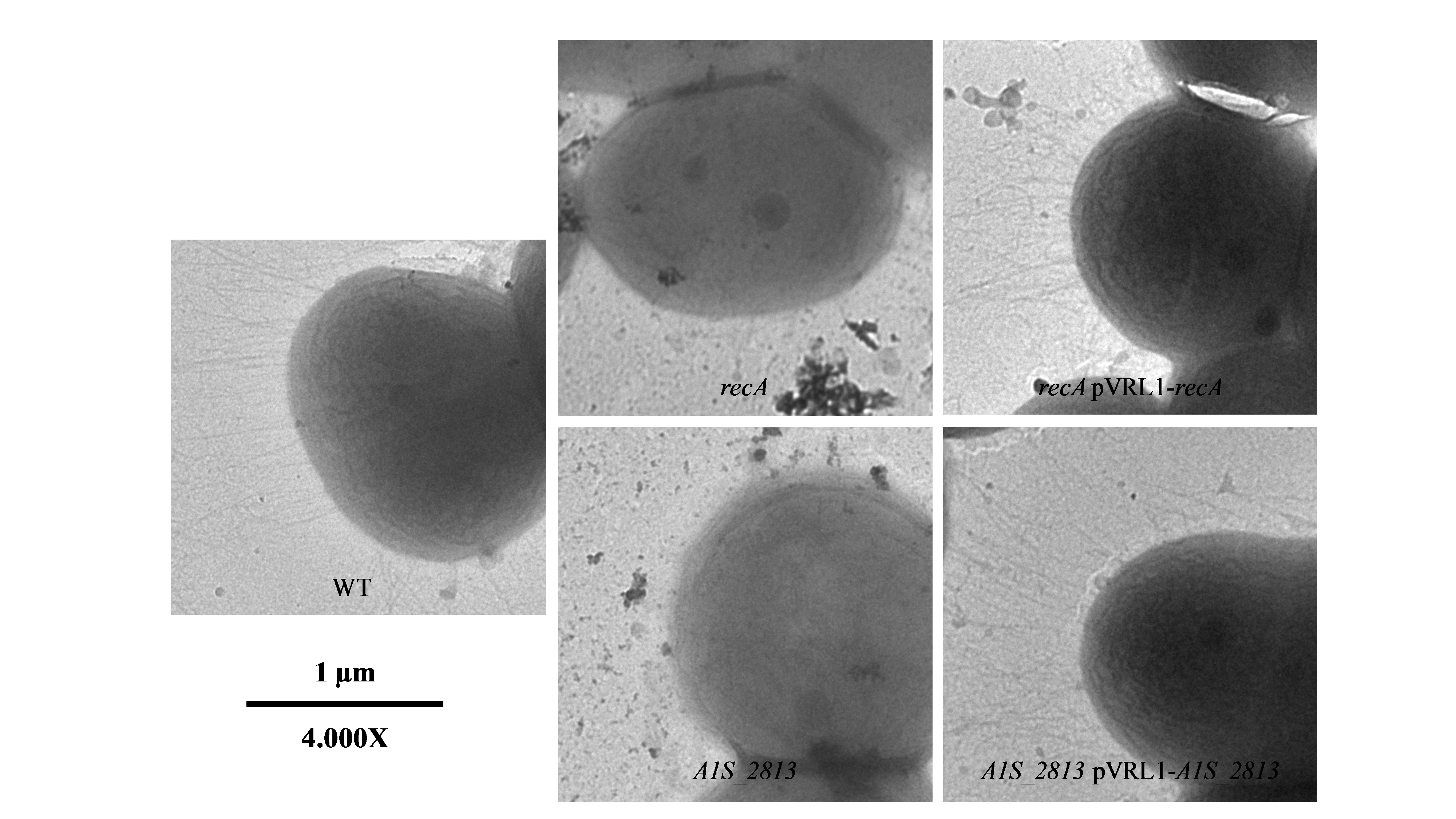

Supplement: Supplemental Material [file kvir-11-01-1748923-s001.zip › Fig. S2.TIF]

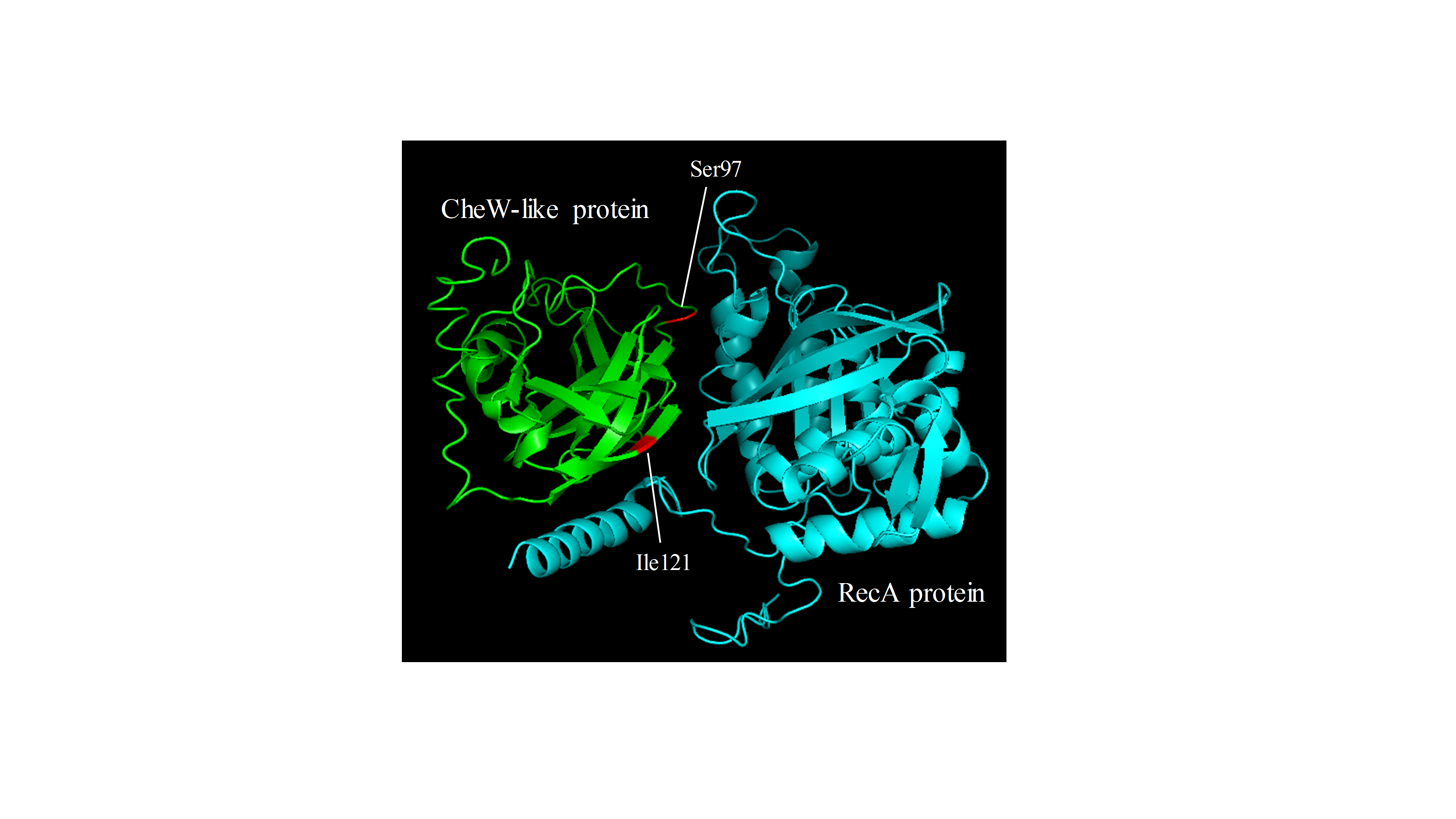

Supplement: Supplemental Material [file kvir-11-01-1748923-s001.zip › Fig. S3.TIF]

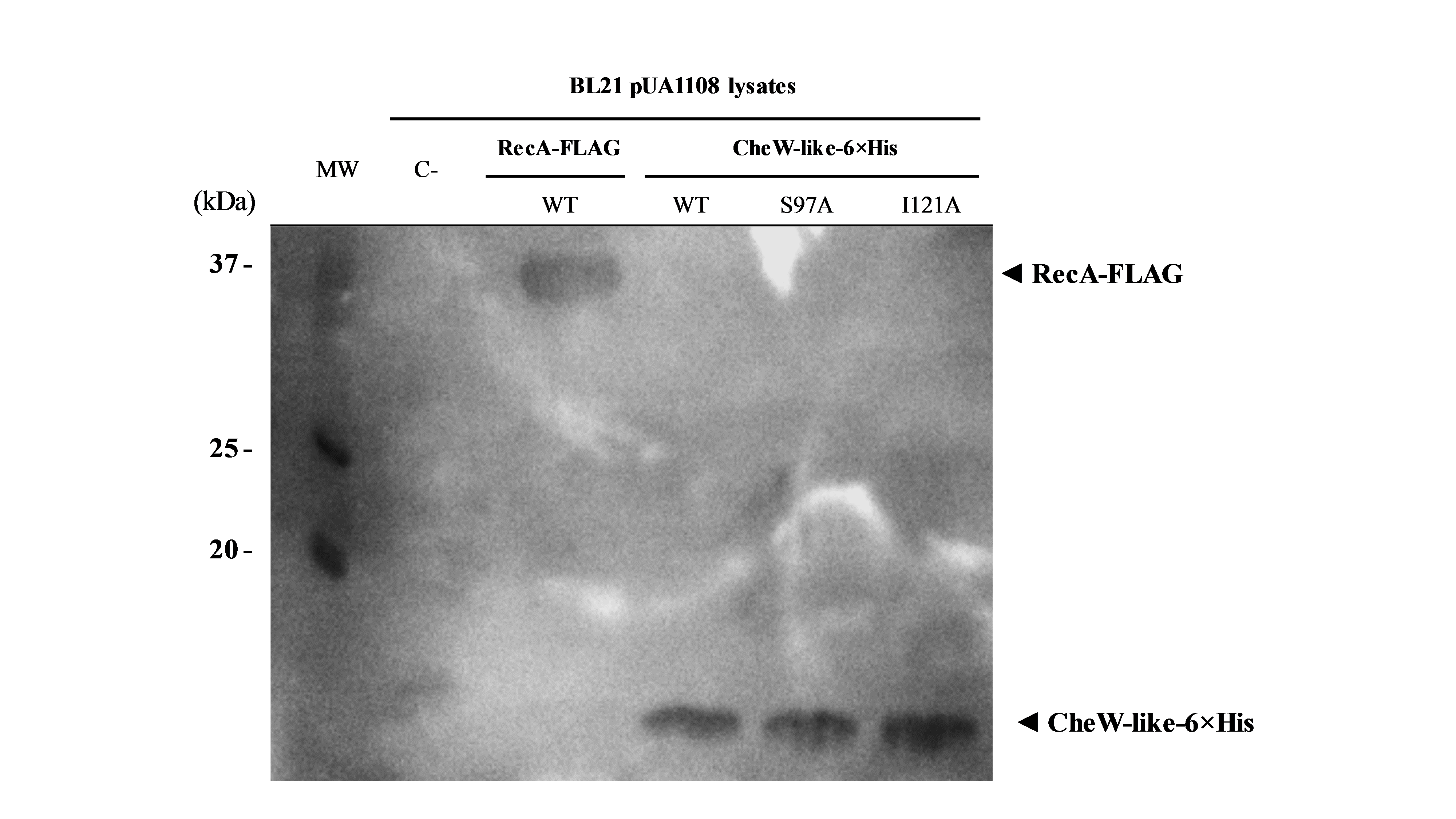

Supplement: Supplemental Material [file kvir-11-01-1748923-s001.zip › Fig. S4.tif]
